# CRISPR/Cas9 mediated specific ablation of *vegfa* in retinal pigment epithelium efficiently regresses choroidal neovascularization

**DOI:** 10.1038/s41598-023-29014-z

**Published:** 2023-03-06

**Authors:** Jinkyu Park, Gang Cui, Hyundong Lee, Han Jeong, Jay Jiyong Kwak, Junwon Lee, Suk Ho Byeon

**Affiliations:** 1 Institute of Vision Research, Department of Ophthalmology, Severance Eye Hospital, Yonsei University College of Medicine, Yonsei-Ro 50-1, Seodaemun-Gu, Seoul, 03722 South Korea; 2 Brain Korea 21 Project for Medical Science, Yonsei University, Seoul, South Korea; 3 Institute of Human Barrier Research, Department of Ophthalmology, Gangnam Severance Hospital, Yonsei University College of Medicine, Eonjuro 211, Gangnam-Gu, Seoul, 06273 South Korea

**Keywords:** Genetic engineering, Molecular biology, Diseases, Gene therapy

## Abstract

The CRISPR/Cas9 system easily edits target genes in various organisms and is used to treat human diseases. In most therapeutic CRISPR studies, ubiquitously expressed promoters, such as CMV, CAG, and EF1α, are used; however, gene editing is sometimes necessary only in specific cell types relevant to the disease. Therefore, we aimed to develop a retinal pigment epithelium (RPE)-specific CRISPR/Cas9 system. We developed a CRISPR/Cas9 system that operates only in retinal pigment epithelium (RPE) by expressing Cas9 under the RPE-specific vitelliform macular dystrophy 2 promoter (pVMD2). This RPE-specific CRISPR/pVMD2-Cas9 system was tested in human retinal organoid and mouse model. We confirmed that this system works specifically in the RPE of human retinal organoids and mouse retina. In addition, the RPE-specific *Vegfa* ablation using the novel CRISPR-pVMD2-Cas9 system caused regression of choroidal neovascularization (CNV) without unwanted knock-out in the neural retina in laser-induced CNV mice, which is a widely used animal model of neovascular age-related macular degeneration. RPE-specific *Vegfa* knock-out (KO) and ubiquitous *Vegfa* KO were comparable in the efficient regression of CNV. The promoter substituted, cell type-specific CRISPR/Cas9 systems can be used in specific ‘target cell’ therapy, which edits genes while reducing unwanted off- ‘target cell’ effects.

## Introduction

The clustered regularly interspaced short palindromic repeat (CRISPR)-Cas (CRISPR-associated) system has considerably contributed to genome editing in diverse species. The CRISPR-Cas system comprises two components: protein (Cas9) and RNA (guide RNA). Cas9 is an endonuclease that creates site-specific double-strand breaks (DSB) in the genomic DNA targeted by guide RNA (gRNA). In contrast to zinc finger nucleases (ZFNs) and transcription activator-like effector nucleases (TALENs), which require substantial engineering to edit the specific genomic DNA target sites, the CRISPR-Cas9 system requires modification of only the gRNA sequence. Therefore, the CRISPR-Cas9 technology has been rapidly and widely adopted by the scientific community to target, edit, or modify the genomes of various cells and organisms^1^.

When we use the CRISPR/Cas9 system for therapeutic purposes, there may be disease relevant target cells to be corrected. If the CRISPR/Cas9 system works in all types of cells, the gene correction occurs even in non-target, unwanted cells. Thus, as a biotechnological tool, cell type-specific gene correction can prevent unwanted off- ‘target cell’ effects. Appropriately engineered CRISPR/Cas9 systems, therefore, are valuable in specific ‘target cell’ therapy, as well as help in investigating the interaction between two or more adjacent cells.

Cell type-specificity of the CRISPR/Cas9-gRNA system is determined by either its delivery or endogenously via controlling Cas9 gene expression. Regarding the gene expression, there may be a way to change the promoter. In most previous CRISPR studies, ubiquitously expressed promoters such as CMV, CAG, and EF1α have been used. In this study, we conferred cell specificity to Cas9 endonucelase by using a cell type-specific promoter, which system can be used irrespective of the various delivery methods.

Age-related macular degeneration (AMD) is the leading cause of blindness in developed countries^2^. Choroidal neovascularization (CNV) is a main pathologic feature of neovascular AMD and is caused by several angiogenic growth factors including vascular endothelial growth factor A (VEGFA)^3^. VEGFA has beens a major therapeutic target for the treatment of neovascular AMD^4^ and the anti-VEGF monoclonal antibodies (e.g., aflibercept, bevacizumab, and ranibizumab) have led to significantly reduced retinal and choroidal neovascularizations^5^. Currently, intravitreous anti-VEGF injection therapy is a mainstay of treatment for neovascular AMD^6,7^. However, anti-VEGF therapies, which require frequent repetitive injections over time to sustain the therapeutic effect, can cause critical adverse effects such as intraocular infection, and impose a great economic burden on the patients^8–10^. Therefore, a single treatment which is effective over the long-term, such as gene therapy could be ideal to suppress pathologic angiogenesis.

We tried to develop retinal pigment epithelium (RPE)-specific CRISPR/Cas9 system and confirmed that this system works specifically in the RPE of human retinal organoids and mouse retina. We then checked the utility of this system in inhibiting laser-induced choroidal neovascularization (CNV), which is a well-established animal model for neovascular AMD, via *Vegfa* ablation.

## Methods

### Construction of lentiviral vector plasmids encoding Cas9 and guide RNA

**Figure 1:**
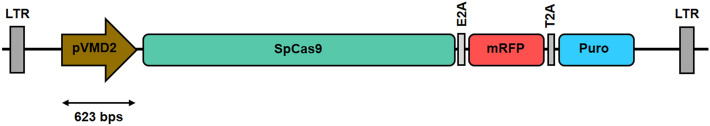
The schematic construct of the pVMD2-SpCas9-E2A-mRFP-T2A-Puro vector. The CMV promoter was substituted with the human VMD2 promoter (623 bps long) which is located at − 585 to + 38 bp of human *VMD2.*

We modified the widely used and ubiquitously expressed Cas9 vector (pCMV-SpCas9-E2A-mRFP-T2A-Puro) for retinal pigment epithelium (RPE)-specific expression by switching CMV promoter (pCMV) with a retinal pigment epithelium (RPE)-specific promoter, the human vitelliform macular dystrophy 2 promoter (pVMD2). The 623 base pairs (bp) long human VMD2 promoter sequence (− 585 to + 38)^11^ was obtained from the genomic DNA of HEK293T (ATCC, VA, USA) by amplification with the forward primer 5′-acgcgtATGCAGAATTCTGTC-3′ and reverse primer 5′-aagcttAAGGTCTGGCGACTAG-3′. The CMV promoter (pCMV) in the pCMV-SpCas9-E2A-mRFP-T2A-Puro vector, which was a gift from Hyongbum Henry Kim (Yonsei University), was substituted with the VMD2 promoter (pVMD2) using the restriction enzymes, MluI and HindIII (Fig. 1).

We used two separate lentiviral vectors, each for the expression of Cas9 and gRNA. To prepare lentiviral vectors, pVMD2-SpCas9-E2A-mRFP-T2A-Puro (pVMD2-Cas9) and pCMV-SpCas9-E2A-mRFP-T2A-Puro (pCMV-Cas9) were subcloned into the lentiCRISPRv2 vector (#52,961; Addgene, MA, USA). The lentiviral vector, LentiGuide-Puro (#52,963, Addgene, MA, USA) was used to express gRNA. The target genes and sequences, protospacer adjacent motif (PAM) sequences, and oligonucleotides for vector subcloning of gRNA are summarized in Supplementary Table 1. Sequences common between the two species were selected for gRNAs targeting mouse *Vegfa* and human *VEGFA*.

### Animals

Adult C57BL/6 J mice (8–10 weeks old, weighing 18–20 g) were used for the study. Mice were maintained in accordance with the Association for Research in Vision and Ophthalmology (ARVO) Statement for the Use of Animals in Ophthalmic and Vision Research. All animal experiments were approved by the Institutional Animal Care and Use Committee (IACUC) of the Yonsei University College of Medicine (IACUC number: 2017–0239). All related methods were performed in accordance with the relevant guidelines and regulation and this study is reported in accordance with ARRIVE guidelines. C57BL/6 mice were maintained under a 12:12-h light/dark cycle.

### Lentivirus production

To produce lentivirus, HEK293T cells were transfected with a plasmid mixture (20 μg) of the transfer plasmids containing the gene of interest, psPAX2 (#12,260, Addgene), and pMD2.G (#12,259, Addgene) at a weight ratio of 4:3:1 using Lipofectamine 2000 (Thermo Fisher Scientific, MA, USA). The supernatant containing the virus was collected at 36 and 60 h after the initial transfection. The supernatant was filtered through a Millex-HV low-protein-binding membrane (0.45 μm pore size, Millipore, MA, USA) and concentrated by centrifugation in Vivaspin 20 (Sartorius, Gottingen, Germany). To obtain a high titer, ultracentrifugation (BECKMAN COULTER) was performed at 24,000 rpm for 2 h at 4 °C. During the ultracentrifuge process, lentiviral particles for in vivo subretinal injection passed through a sucrose cushion^12^. After ultracentrifugation, the supernatant was completely removed and the remaining viral pellets were resuspended in 200 μL PBS, left undisturbed overnight at 4 °C, and stored at − 80 °C. The lentiviral titer was measured using the NuceloSpin RNA virus Kit (TaKaRa, Shiga, Japan) and Lenti-X qRT-PCR Titration Kit (TaKaRa, Shiga, Japan), according to the manufacturer’s instructions.

### Sub-retinal injection and in vivo choroidal neovascularization model

Cas9 and gRNA were delivered into the sub-retinal space using methods in previous experiments^13,14^, following which the mice were subjected to laser-induced choroidal neovascularization (CNV).

After mice were anesthetized, pupils were dilated with an eye drop containing phenylephrine (0.5%) and tropicamide (0.5%). Before subretinal injection, a small incision site was made slightly posterior to the limbus at 11 o’clock position using a 30-gauge 1/2″ needle (Supplementary Fig. 1A). A 33-gauge needle of sub-microliter injection system (World Precision Instruments, FL, USA) was passed through the incision site. The subretinal injection of lentivirus was performed at inferior area of fundus. After slow injection to ensure proper distribution of the injected solution, the formation of a diffuse bleb was confirmed, and the needle was gently withdrawn (Supplementary Fig. 1B).

Two microliters of a solution containing 8 × 10^10^ viral genomes (vg)/mL of lentivirus [comprising 1 μL of pVMD2-Cas9 or pCMV-Cas9 and 1 μL of gRNA targeting *Vegfa* (*Vegfa-*gRNA*)* or *Rosa26 (Rosa26-*gRNA)] was delivered into the mouse subretinal space. One week after lentiviral transduction, choroidal neovascularization was induced using laser photocoagulation^15^, by an argon laser system (NIDEK, CA, USA) using 532 nm wavelength with 50 μm spot size, 100 mW power, and 100 ms exposure time. Four laser burns were made at a certain distance from each other around the optic nerve at the regions where the blebs were formed by the subretinal injection (Supplementary Fig. 1C). Only burns that produced bubbles without hemorrhage were included in the analysis.Only CNV lesions within the subretinal injected area were included in the analysis (Supplementary Fig. 1D). Positive controls consisted of mice in which aflibercept was injected intravitreally at the same time as CNV induction. One week after CNV induction, tissues were harvested, and CNV size was evaluated.

### Retinal organoid differentiation and lentiviral transduction

Retinal organoids were differentiated from human embryonic stem cells (hESCs), H9, following a previously described protocol with minor modifications^16,17^. After 130–160 days of differentiation, retinal organoids containing RPE spheres were selected for transduction experiments. We transduced a total of 8 × 10^9^ copies of lentivirus (4 × 10^9^ each of Cas9-containing and gRNA-containing lentiviruses) per organoid in 200 μL of Long-Term Retina medium with 8 μg/mL Polybrene (Sigma-Aldrich, Missouri, USA). Long-Term Retina medium (300 μL) was added 24 h after transduction. After 48 h incubation, the transduced retinal organoids were rinsed in 3X PBS and then maintained in an Long-Term Retina medium without lentivirus. Two weeks after lentiviral transduction, fluorescence images were analyzed and cells were harvested. RPE spheres and neural retinas (NR) were manually isolated and used for analysis.

### Immunohistochemistry of mouse retina and retinal organoid

For analyzing cross-sectional images of mouse NR and RPE, the eye was enucleated, the cornea was punctured, and the eye was fixed for 1 h in 4% paraformaldehyde in PBS. The fixed eyes were cryopreserved by sinking in 15% sucrose in PBS, followed by equilibration in 30% sucrose. For analyzing cross-sectional images of retinal organoid, the organoid was fixed in 1% paraformaldehyde in PBS. The fixed organoids were cryopreserved by sinking in 10% sucrose in PBS, followed by equilibration in 30% sucrose. Mouse eyes and retinal organoids were embedded in OCT compound and snap-frozen in liquid nitrogen. Samples were cut into 7-μm thick sections using a cryostat. Cryosections of mouse retina were immunostained with the primary antibody, anti-RFP antibody (Invitrogen, MA, USA), and secondary antibody, Alexa Fluor™ 594 anti-rabbit IgG (Invitrogen, MA, USA). Retinal organoids were immunostained with various markers of retinal cell types according to the developmental stage. The antigen, host species, used concentration, and supplier of the primary antibody used are listed in Supplementary Table 2.

### Angiography of mouse retina

To evaluate CNV size, FITC-dextran was intravenously injected 5 min before sacrifice. The enucleated eyes were fixed in 4% paraformaldehyde for 1 h at room temperature. The RPE complex (RPE/choroid/sclera) was flat-mounted and viewed under a fluorescent microscope (BX43; Olympus, Tokyo, Japan). The CNV area was measured using ImageJ software (NIH) by blinded observers. An average of 3–4 CNV areas per eye were analyzed. Statistical analysis was performed for each CNV lesion, not for each eye.

### Targeted deep sequencing

On-target and off-target regions were amplified from the genomic DNA using specific primers (Supplementary Table 3) for targeted deep sequencing. Deep-sequencing libraries were generated using PCR. TruSeq HT dual-index primers were used to label the samples. Pooled libraries were subjected to paired-end sequencing using HiSeq X (Illumina, CA, USA). Indels around the site 3 bp upstream of the PAM sequence were considered mutations resulting from Cas9 activity. Indel frequencies were calculated using bioinformatic tools^18^.

### Measurement of VEGF in mouse vitreous samples

Vitreous samples were collected using glass micropipettes. A micropipette puller is used to pull the tips of standard glass pipettes. Mice were anesthetized and the tip was inserted through the sclera 2 mm posterior to the limbus. Suction was activated by stepping on the foot pedal with a pump in reverse mode and vitreous was slowly aspirated, allowing collection of as much as 4 μL. Samples were immediately placed on ice, and the samples were centrifuged at 6000 rpm for 30 sec^19^. 4 μL vitreous humor (from a single eye) was diluted in 6 μL RIPA buffer (Rockland Immunochemicals, PA, USA) containing protease inhibitor (Quartett, Berlin, Germany). VEGF protein levels of the vitreous humor samples were measured using a mouse VEGF Quantikine ELISA kit (R&D Systems, Minneapolis, MN, USA), according to the manufacturer’s instructions.

### Statistical analysis

All group results are expressed as mean ± standard error of the mean (S.E.M.). Mean comparisons between groups were performed using the two-tailed Student’s t-test or one-way ANOVA and Tukey post-hoc analyses for multiple groups. Statistical significance is denoted with *(*P* < 0.05), **(*P* < 0.01), ***(*P* < 0.001) in the figures and figure legends. The statistics were analyzed using SPSS software (IBM SPSS Statistics, Armonk, NY, USA).

## Results

**Figure 2:**
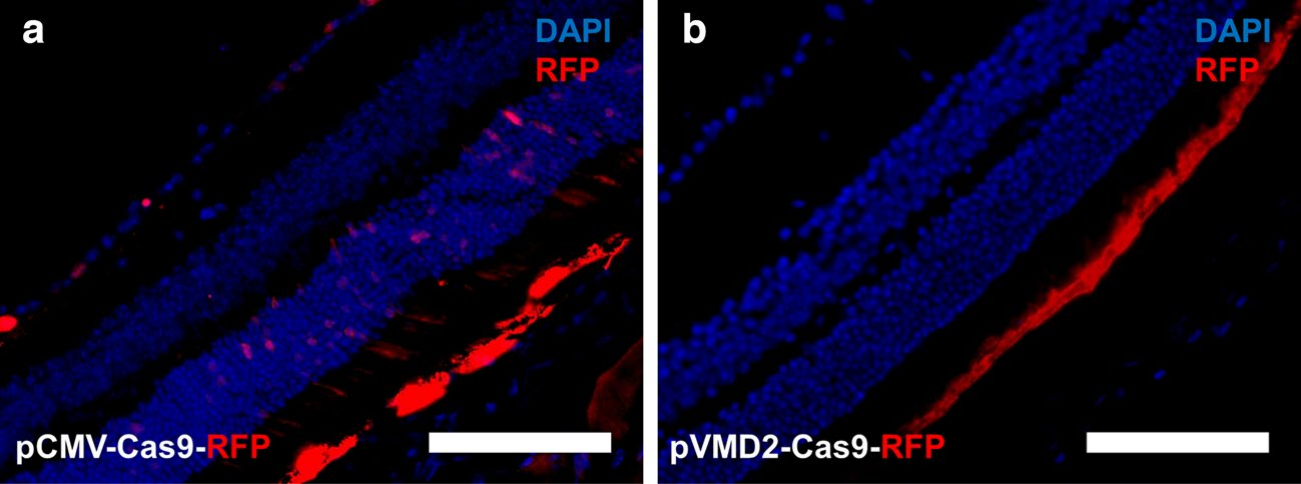
Expression patterns of pCMV-SpCas9-RFP and pVMD2-SpCas9-RFP in mice. (**a**) The pCMV vector expressed RFP both in the RPE and neural retina; (**b**) whereas the pVMD2 vector expressed RFP only in RPE (Scale bar = 100 µm).

We first analyzed the effect of either promoter–pCMV and pVMD2-on cell-specificity of Cas9 expression. Lentiviruses carrying pCMV-SpCas9-E2A-mRFP-T2A-Puro (pCMV-SpCas9) and pVMD2-SpCas9-E2A-mRFP-T2A-Puro (pVMD2-SpCas9) vectors were transduced into the mice subretinal space via subretinal injection. The expression patterns of these two vectors were determined by the expression of red fluorescent protein (RFP). We found that while the RFP was expressed in the RPE and neural retina for pCMV-SpCas9 lentivirus (Fig. 2a), the pVMD2-SpCas9 lentivirus expressed RFP only in the RPE (Fig. 2b).

**Figure 3:**
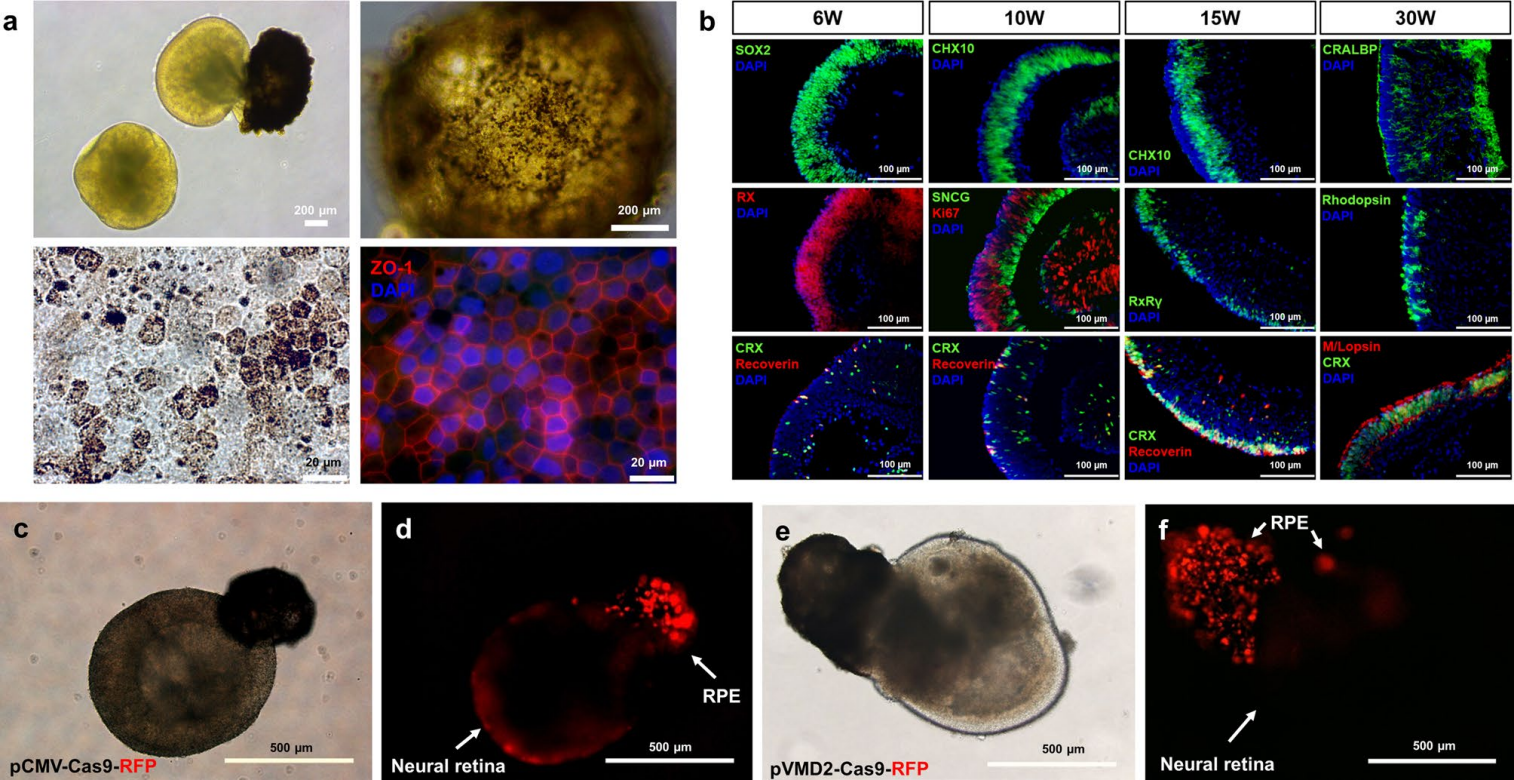
Expression patterns of pCMV-SpCas9-RFP and pVMD2-SpCas9-RFP in human retinal organoids. (**a**) RPE characteristics of pigmentation and hexagonality were confirmed in a 3D sphere and 2D monolayer status (**b**) Retinal organoids express various retinal markers being appropriate to normal developmental stages. A bright-field image and fluorescent image of the human retinal organoid are displayed in pairs (**c**,**d**; **e**,**f**). (**c**,**d**) The pCMV vector expressed RFP both in the RPE and neural retina; (**e**,**f**) whereas the pVMD2 vector expressed RFP only in RPE [**c**,**e** = bright-field image; **d**,**f** = fluorescent image]. Each protein marks specific structure or cell types as follows: ZO-1: Tight junction; SOX2: Neural progenitor cell; RX: Retinal progenitor cell; CRX: Photoreceptor progenitor cell; Recoverin: Photoreceptor precursor cell; CHX10: Neural retinal progenitor cell; SNCG: Retinal ganglion cell; Ki67: Proliferating cell; RxRγ: Cone precursor cell; CRALBP: Muller glia cell; Rhodopsin: Rod photoreceptor cell; M/L opsin: M/L cone photoreceptor.

The expression patterns of lentiviruses carrying pCMV-SpCas9 and pVMD2-SpCas9 vectors were then tested in human retinal organoids with RPE spheres. When human retinal organoids were differentiated from human embryonic stem cell line of H9, RPE spheres usually co-occur in about 20% of the retinal organoids. The RPE characteristics of pigmentation and hexagonality were confirmed even in a 3D sphere state and in monolayer 2D culture of dissociated RPE spheres (Fig. 3a) Retinal organoids express various retinal markers being appropriate to normal developmental stages (Fig. 3b). Like the in vivo results obtained in mouse sub-retina, the pCMV-spCas9 vector expressed RFP in both RPE and NR (Fig. 3c,d), whereas the pVMD2-SpCas9 vector expressed RFP only in the RPE (Fig. 3e,f).

**Figure 4:**
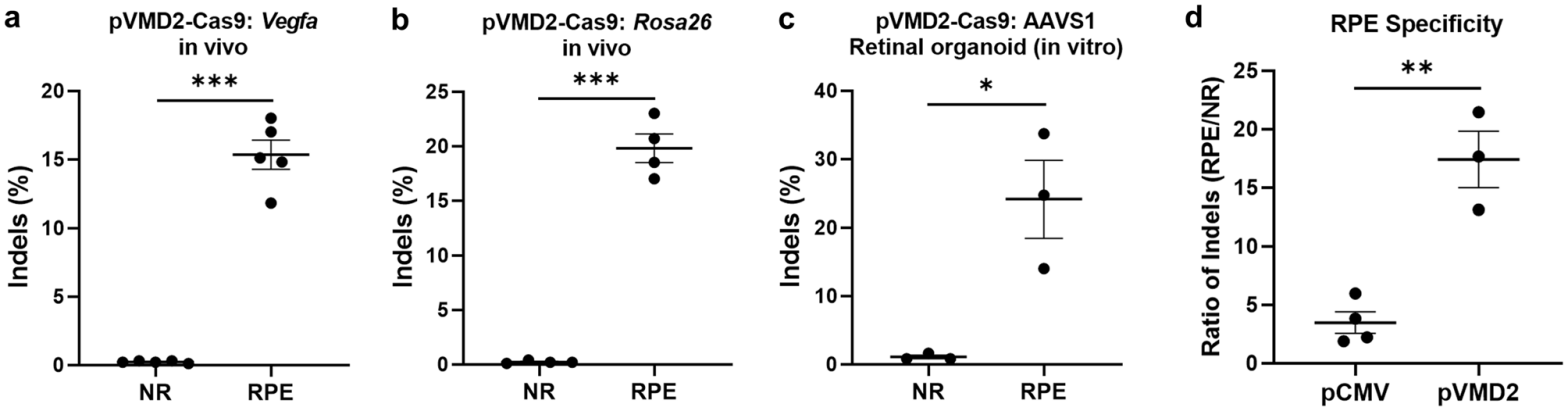
RPE-specific gene editing by pVMD2-SpCas9. (**a**,**b**) Subretinal injection of two lentiviruses containing pVMD2-SpCas9 and mouse *Vegfa-* or *Rosa26*-targeting guide RNA (gRNA) was performed. Generated indels (Insertion and deletion) efficiencies were significantly higher in RPE than in neural retina (NR) (*Vegfa*, *p* = 0.005; *Rosa26*, *p* < 0.001); (**c**) When lentiviruses of pVMD2-SpCas9 and human *AAVS1*-targeting gRNA were transduced into human retinal organoid containing RPE sphere, indel efficiencies were significantly higher in RPE than in NR (*p* = 0.016); (**d**) The ratio of indels generated in RPE compared to indels generated in the neural retina (NR) was defined as RPE specificity. pVMD2-SpCas9 showed significantly higher RPE specificity than pCMV-SpCas9 (*p* = 0.002).

We next investigated the gene editing effect of subretinal injection of lentiviruses carrying pVMD2-SpCas9 and *Vegfa-* or *Rosa26-*gRNA on indel (insertion and deletion) mutations in the RPE and NR. Expression of *Vegfa-* and *Rosa26-*gRNA alongside pVMD2-SpCas9 induced significantly higher indel frequency in RPE compared to NR [%, RPE vs. NR; *Vegfa*, 15.34 ± 1.07 (*n* = 5) vs. 0.22 ± 0.04 (*n* = 5), *p* < 0.0001 (Fig. 4a); *Rosa26,* 19.80 ± 1.31 (*n* = 4) vs. 0.23 ± 0.13 (*n* = 4), *p* < 0.0001 (Fig. 4b)]. Only one site with a mismatch of one or two bases to the target sequence of *Vegfa*-gRNA was found in the whole mouse genome and no off-target effect on that site was observed by targeted deep sequencing. Similar results were obtained with lentiviral transduction of pVMD2-SpCas9 and human Adeno-Associated Virus Integration Site 1 (*AAVS1*)-gRNA into human retinal organoid. Indel frequency was significantly higher in RPE compared to NR [%, RPE vs. NR; *AAVS1*, 24.13 ± 5.69 (*n* = 3) vs. 1.07 ± 0.27 (*n* = 3), *p* = 0.016, (Fig. 4c)]. The ratio of indels generated in RPE compared to indels generated in NR (Indel_RPE_/Indel_NR_) was defined as RPE specificity. pVMD2-SpCas9 showed significantly higher RPE specificity compared to pCMV-SpCas9 [Indel_RPE_/Indel_NR_, pVMD2-SpCas9 vs. pCMV-SpCas9, 17.40 ± 2.41 (*n* = 3) vs. 3.48 ± 0.93 (*n* = 4), *p* = 0.002 (Fig. 4d)].

**Figure 5:**
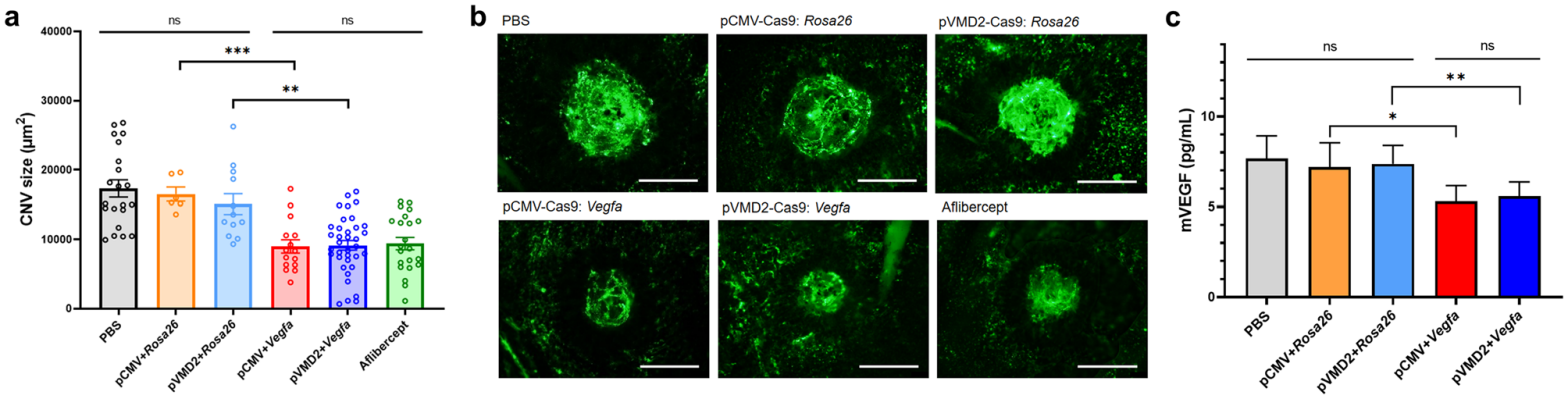
RPE-specific *Vegfa* ablation efficiently regresses choroidal neovascularization. (**a**,**b**) When evaluating the therapeutic effect of pVMD2-SpCas9/ pCMV-SpCas9 and mouse *Vegfa*-targeting gRNA through subretinal injection in laser-induced CNV mice model, pVMD2-SpCas9 and *Vegfa*-targeting gRNA significantly regressed CNV compared to negative controls and was comparable to pCMV-SpCas9 and *Vegfa*-targeting gRNA and aflibercept. [Scale bar = 200 µm (**b**)]; (**c**) The amount of mouse VEGF protein in vitreous was significantly decreased compared to controls in both Cas9 and *Vegfa*-targeting gRNA groups.

We evaluated the therapeutic effect of pVMD2-SpCas9 and *Vegfa*-gRNA on regression in laser-induced CNV in vivo. Treatment with *Rosa26*-gRNA in combination with either pCMV-SpCas9 or pVMD2-SpCas9 did not significantly reduce CNV size compared to that of PBS-injected negative control [CNV size, µm^2^; PBS, 17,335 ± 1231 (*n* = 21); vs. pCMV-Cas9 and *Rosa26*-gRNA, 16,517 ± 1007 (*n* = 6), *p* = 0.999; vs. pVMD2-Cas9 and *Rosa26*-gRNA, 15,074 ± 1493 (*n* = 12), *p* = 0.734]. Transduction with pVMD2-SpCas9 and *Vegfa*-gRNA caused significant regression in CNV compared to the control [pVMD2-SpCas9 and *Vegfa*-gRNA, 9159 ± 703 (*n* = 36) vs. pVMD2-SpCas9 and *Rosa26*-gRNA, 15,074 ± 1493 (*n* = 12), *p* = 0.002], as being compararble to transduction with pCMV-SpCas9 and *Vegfa*-gRNA and clinically used intravitreal aflibercept [vs. pCMV-SpCas9 and *Vegfa*-gRNA, 8999 ± 971 (*n* = 15), *p* = 1.000; vs. Aflibercept, 9405 ± 890 (*n* = 22), *p* = 1.000] (Fig. 5a,b).

The VEGF protein expression was significantly decreased in vitreous of pCMV-SpCas9 and *Vegfa*-gRNA group and pVMD2-SpCas9 and *Vegfa*-gRNA group compared to PBS-treated group [mVEGF, pg/mL; PBS, 7.67 ± 0.48 (n = 7); vs. pCMV-Cas9 and *Vegfa*-gRNA, 5.29 ± 0.36 (*n* = 6), *p* = 0.003; vs. pVMD2-Cas9 and *Vegfa*-gRNA, 5.58 ± 0.28 (*n* = 8), *p* = 0.002].In the case of pCMV Cas9 and pVMD2 Cas9, targeting *Rosa26* and *Vegfa*, respectively, there were significant differences [pCMV-Cas9 and *Rosa26*-gRNA, 7.18 ± 0.55 (*n* = 6); vs. pCMV-Cas9 and *Vegfa*-gRNA, *p* = 0.016; pVMD2-Cas9 and *Rosa26*-gRNA, 7.36 ± 0.39 (*n* = 7); vs. pVMD2-Cas9 and *Vegfa*-gRNA, *p* = 0.002]. There was no difference between the pCMV-SpCas9 and pVMD2-SpCas9 groups (*p* = 0.538) (Fig. 5c).

## Discussion

The two components of the CRISPR/Cas9 system-Cas9 endonuclease and gRNA–are both required for its activity. Thus, assigning cell-specificity to either one is enough to target the CRISPR/Cas9 system to specific cells. Here, we constructed an RPE-specific CRISPR/Cas9 system by controlling Cas9 endonuclease expression via promoter substitution.

We compared the effect of switching pCMV (ubiquitous promoter) and pVMD2 (RPE-specific promoter) on the expression pattern of Cas9 endonuclease and the gene editing effect of the indel frequency in RPE and NR using in vivo and in vitro experiments. Compared to vectors containing pCMV-Cas9, those with pVMD2-Cas9 expressed specifically in the RPE and showed fivefold higher RPE specificity with respect to the indel frequency. Some indels observed in the NR with pVMD2-Cas9 transduction could be due to the inevitable contamination occurring during the manual separation of RPE and NR. Actual RPE specificity of pVMD2-Cas9 may be higher than that experimentally observed.

In a previous report, RPE specificity was conferred by changing the lentivirus envelope. On subretinal injection, lenti-VSVG transduced RPE and photoreceptors, whereas transgene expression was restricted to RPE cells using lentivirus with Mokola envelope^20^. RPE specific expression was also confirmed in the delivery of DNA using various nanoparticles^21–23^. Also, as in our report, there have been reports of inducing cell-specific expression through promoter substitution, and the VMD2 promoter has been widely used for RPE-specific gene therapy and CNV regression^12,24,25^.

Laser-induced CNV model is a well-established animal model of AMD and there have been various studies that CNV was successfully regressed through *Vegfa* KO using CRISPR. Jin-soo Kim group showed successful CNV regression through *Vegfa* KO through subretinal injection of ribonucleoprotein (RNP) form of ‘Cas9 protein and gRNA ribonucleoprotein’ and cationic lipid^14^. The group used the same gRNA that was used in our report, and showed 25% Vegfa KO in in vivo RPE, which was higher than our report of 15%. In the same group, *Vegfa* KO and regression of CNV was confirmed through intravitreal injection of AAV using a small Cas9 of Campylobacter jejuni Cas9 and different programmable endonuclease of Lachnospiraceae bacterium Cpf1^13,26^. Thomas J. Corydon group reported that the transduction occurred only in RPE, and indel was identified up to 84% of transduced RPE in vivo through subretinal injection of lentivirus^27^. Since only the transduced RPE was analyzed, direct comparison with the indel efficiency in our report was difficult; however, the result was different from ours confirming partial transduction of the photoreceptor. More recently, advanced strategies of delivery tool and CRISPR, using such as lentiviral delivery of co-packaged Cas9 mRNA and a guide RNA^28^, RNP Complexes Containing Cas9 Protein and Modified sgRNAs^29^, and paired guide RNAs^30^, showed effectiveness in CNV regression. However, there has been no report on RPE specific gene editing using promoter substitution in in vivo or in vitro retina.

The VEGF pathway is the most important pathomechanism of CNV; VEGF is expressed significantly in RPE and Müller cells and those cells could be the main source of VEGF^31,32^. VEGF also acts as a survival factor in other retinal cell types including Müller cells, photoreceptors, and retinal ganglion cells^33,34^; thus, the long-term side effects of *VEGF* ablation in the NR may occur. We attempted to reduce the off- ‘target cell’ effect while achieving the therapeutic goal through RPE-specific *Vegfa* ablation. In a laser-induced CNV mouse model, compared to mock treatment, RPE-specific *Vegfa* ablation under the pVMD2 showed efficient CNV regression, which is comparable to the clinical use of aflibercept and ubiquitous *Vegfa* ablation (under pCMV). This provides additional and indirect evidence that the main source of VEGFA in CNV is the RPE. Sufficient therapeutic objectives could be achieved without genetic modification in non-target cells and other potential risks.

The biggest drawback of our study is the use of lentivirus. Although AAV could not be used due to limitation of cargo size, it would be advantageous to use AAV considering better feasibility in clinical applications as well as high and evenly transduction efficiency for both RPE and photoreceptor.

In summary, we developed a CRISPR/Cas9 system in which Cas9 is expressed under a promoter specific to RPE, the human VMD2 promoter. We confirmed that this CRISPR/pVMD2-Cas9 showed the specific expression and gene editing in RPE cells in both in vivo (mouse) and in vitro (human retinal organoid) models. We also confirmed that RPE-specific *Vegfa* gene knockout, using the CRISPR/pVMD2-Cas9 developed here, efficiently and sufficiently causes regression of CNV in a laser-induced choroidal neovascularization (CNV) mouse model.

## Supplementary Information

Supplementary Information 1.

Supplementary Information 2.

## Data Availability

All data generated or analysed during this study are included in this published article (and its Supplementary Information files). The datasets generated during and/or analysed during the current study are available from the corresponding author on reasonable request.

## References

[1] Doudna JA (2020). The promise and challenge of therapeutic genome editing. Nature.

[2] Fleckenstein M (2021). Age-related macular degeneration. Nat. Rev. Dis. Primers.

[3] Ferrara N (2010). Vascular endothelial growth factor and age-related macular degeneration: from basic science to therapy. Nat. Med..

[4] Leung DW, Cachianes G, Kuang WJ, Goeddel DV, Ferrara N (1989). Vascular endothelial growth-factor is a secreted angiogenic mitogen. Sci. 1979.

[5] Amoaku WM (2015). Defining response to anti-VEGF therapies in neovascular AMD. Eye Lond.

[6] Group CR (2011). Ranibizumab and bevacizumab for neovascular age-related macular degeneration. N. Engl. J. Med..

[7] Schmidt-Erfurth U (2014). Intravitreal aflibercept injection for neovascular age-related macular degeneration: Ninety-six-week results of the VIEW studies. Ophthalmology.

[8] Funk M (2009). Neovascular age-related macular degeneration: intraocular cytokines and growth factors and the influence of therapy with ranibizumab. Ophthalmology.

[9] Sawada O (2010). Aqueous vascular endothelial growth factor after intravitreal injection of pegaptanib or ranibizumab in patients with age-related macular degeneration. Retina.

[10] Yiu G, Tieu E, Nguyen AT, Wong B, Smit-McBride Z (2016). Genomic disruption of VEGF-A expression in human retinal pigment epithelial cells using CRISPR-Cas9 endonuclease. Invest. Ophthalmol. Vis. Sci..

[11] Esumi N, Oshima Y, Li Y, Campochiaro PA, Zack DJ (2004). Analysis of the VMD2 promoter and implication of E-box binding factors in its regulation. J. Biol. Chem..

[12] Askou AL (2015). Multigenic lentiviral vectors for combined and tissue-specific expression of miRNA- and protein-based antiangiogenic factors. Mol. Ther. Methods Clin. Dev..

[13] Koo T (2018). CRISPR-LbCpf1 prevents choroidal neovascularization in a mouse model of age-related macular degeneration. Nat. Commun..

[14] Kim K (2017). Genome surgery using Cas9 ribonucleoproteins for the treatment of age-related macular degeneration. Genome Res..

[15] Lambert V (2013). Laser-induced choroidal neovascularization model to study age-related macular degeneration in mice. Nat. Protoc..

[16] Eldred KC (2018). Thyroid hormone signaling specifies cone subtypes in human retinal organoids. Science.

[17] Wahlin KJ (2017). Photoreceptor outer segment-like structures in long-term 3D retinas from human pluripotent stem cells. Sci. Rep..

[18] Park J, Lim K, Kim JS, Bae S (2017). Cas-analyzer: An online tool for assessing genome editing results using NGS data. Bioinformatics.

[19] Fortmann SD, Lorenc VE, Shen J, Hackett SF, Campochiaro PA (2018). Mousetap, a novel technique to collect uncontaminated vitreous or aqueous and expand usefulness of mouse models. Sci. Rep..

[20] Auricchio A (2001). Exchange of surface proteins impacts on viral vector cellular specificity and transduction characteristics: The retina as a model. Hum. Mol. Genet..

[21] Bejjani RA (2005). Nanoparticles for gene delivery to retinal pigment epithelial cells. Mol. Vis..

[22] Mitra RN (2014). Synthesis and characterization of glycol chitosan DNA nanoparticles for retinal gene delivery. ChemMedChem.

[23] Sun D (2017). Targeted multifunctional lipid ECO plasmid DNA nanoparticles as efficient non-viral gene therapy for leber's congenital amaurosis. Mol. Ther. Nucleic Acids.

[24] Askou AL (2019). Suppression of choroidal neovascularization by AAV-based dual-acting antiangiogenic gene therapy. Mol. Ther. Nucleic Acids.

[25] Wang H, Kunz E, Stoddard GJ, Hauswirth WW, Hartnett ME (2019). Optimal inhibition of choroidal neovascularization by scAAV2 with VMD2 promoter-driven active Rap1a in the RPE. Sci. Rep..

[26] Kim E (2017). In vivo genome editing with a small Cas9 orthologue derived from Campylobacter jejuni. Nat. Commun..

[27] Holmgaard A (2017). In vivo knockout of the *vegfa* gene by lentiviral delivery of CRISPR/Cas9 in mouse retinal pigment epithelium cells. Mol. Ther. Nucleic Acids.

[28] Ling S (2021). Lentiviral delivery of co-packaged Cas9 mRNA and a *Vegfa*-targeting guide RNA prevents wet age-related macular degeneration in mice. Nat. Biomed. Eng..

[29] Holmgaard AB (2021). Targeted knockout of the *vegfa* gene in the retina by subretinal injection of RNP complexes containing Cas9 protein and modified sgRNAs. Mol. Ther..

[30] Chung SH (2022). CRISPR-based VEGF suppression using paired guide RNAs for treatment of choroidal neovascularization. Mol. Ther. Nucleic Acids.

[31] Kwak N, Okamoto N, Wood JM, Campochiaro PA (2000). VEGF is major stimulator in model of choroidal neovascularization. Invest. Ophthalmol. Vis. Sci..

[32] Robbins SG, Conaway JR, Ford BL, Roberto KA, Penn JS (1997). Detection of vascular endothelial growth factor (VEGF) protein in vascular and non-vascular cells of the normal and oxygen-injured rat retina. Growth Factors.

[33] Saint Geniez M (2008). Endogenous VEGF is required for visual function: evidence for a survival role on müller cells and photoreceptors. PLoS ONE.

[34] Beck M, Munk MR, Ebneter A, Wolf S, Zinkernagel MS (2016). Retinal ganglion cell layer change in patients treated with anti-vascular endothelial growth factor for neovascular age-related macular degeneration. Am. J. Ophthalmol..

