# Supplementary figures and images for: CRISPR/Cas9 mediated specific ablation of vegfa in retinal pigment epithelium efficiently regresses choroidal neovascularization
Source: Sci Rep. 2023 Mar 6;13:3715. doi: 10.1038/s41598-023-29014-z (PMC9988861; doi:10.1038/s41598-023-29014-z)

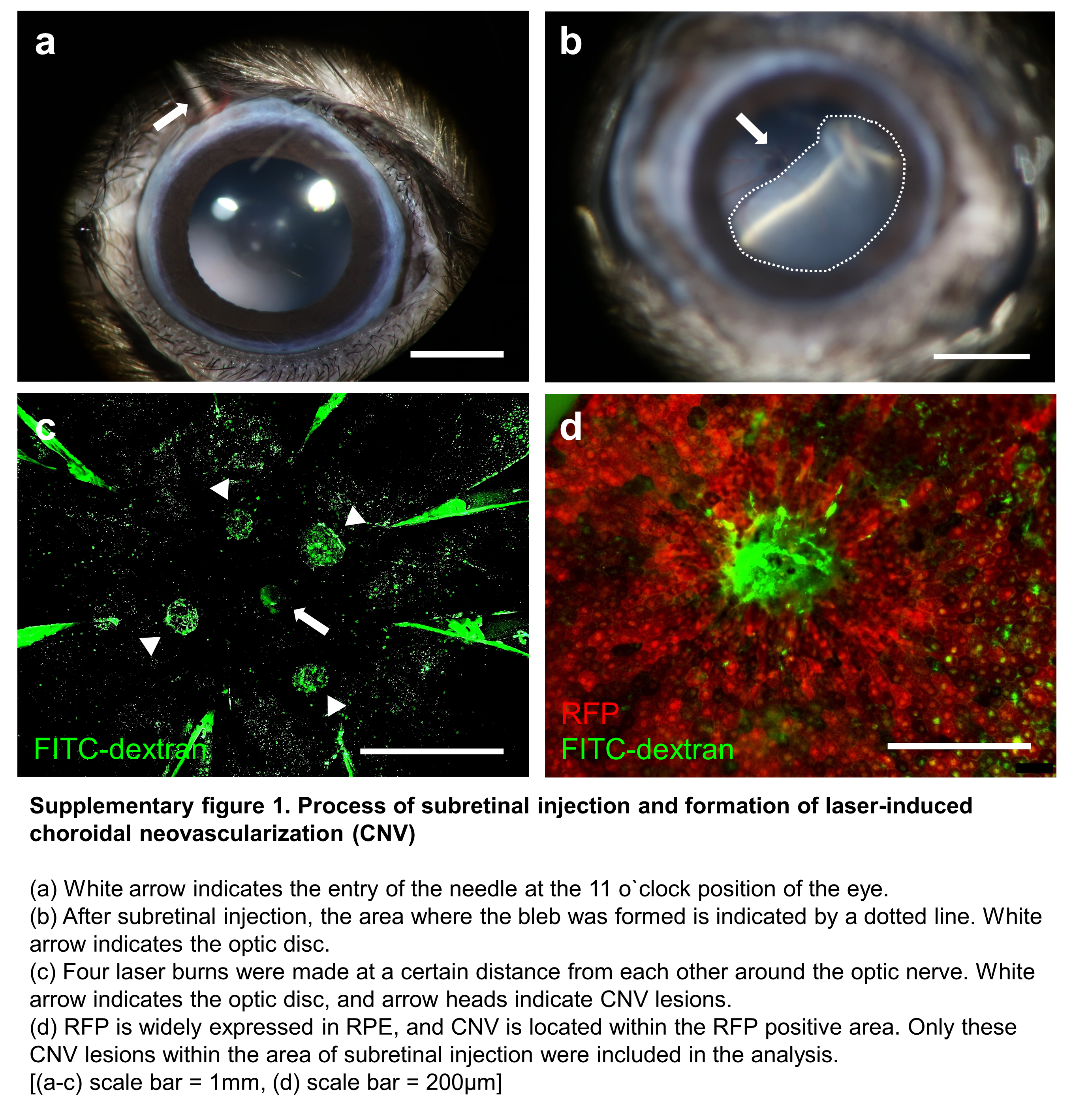

Supplement: Supplementary file 1 — Supplementary Information 1. [file 41598_2023_29014_MOESM1_ESM.tif]
