# Supplementary material for: CRISPR/Cas9 mediated specific ablation of vegfa in retinal pigment epithelium efficiently regresses choroidal neovascularization
Source: Sci Rep. 2023 Mar 6;13:3715. doi: 10.1038/s41598-023-29014-z (PMC9988861; doi:10.1038/s41598-023-29014-z)
Supplement: Supplementary file 2 — Supplementary Information 2. [file 41598_2023_29014_MOESM2_ESM.docx]

**Supplementary Table 1.** Target genes and target sequences and used oligonucleotides for subcloning of guide RNA

| **Target gene** | **Target sequences** | **Forward oligo (5’ to 3’)** | **Reverse oligo (5’ to 3’)** |
| --- | --- | --- | --- |
| *VEGFA* (human) & *Vegfa* (mouse) | CTCCTGGAAGATGTCCACCA **GGG** | caccgCTCCTGGAAGATGTCCACCA | aaacTGGTGGACATCTTCCAGGAGc |
| *AAVS1* (human) | GGGGCCACTAGGGACAGGAT **TGG** | caccgGGGGCCACTAGGGACAGGAT | aaacATCCTGTCCCTAGTGGCCCCc |
| *Rosa26* (mouse) | GGCGGTCCTCAGAAGCCAGG **AGG** | caccgGGCGGTCCTCAGAAGCCAGG | aaacCCTGGCTTCTGAGGACCGCCc |

**Supplementary Table 2.** Antibodies used for immunostaining of retinal organoids

| Antigen | Host species | Used concentration | Supplier |
| --- | --- | --- | --- |
| RFP | Mouse/Rabbit | 1:500 | Invitrogen (MA5-15257, R10367) |
| ZO-1 | Rabbit | 1:200 | Thermofisher (61-7300) |
| SOX2 | Mouse | 1:100 | Santa Cruz Biotechnology  (sc-365964) |
| RX | Rabbit | 1:300 | Abcam (ab23340) |
| CHX10 | Mouse | 1:200 | Santa Cruz Biotechnology  (sc-365519) |
| CRX | Mouse | 1:1000 | Abnova (H00001406-M02) |
| Recoverin | Rabbit | 1:1000 | Millipore (AB5585) |
| SNCG | Mouse | 1:500 | Abnova (H00006623-M01A) |
| Ki67 | Rabbit | 1:500 | Abcam (ab15580) |
| RxR**γ** | Mouse | 1:300 | Santa Cruz Biotechnology  (sc-514134) |
| CRALBP | Mouse | 1:500 | Abcam (ab15051) |
| Rhodopsin | Mouse | 1:1000 | Sigma-Aldrich (R5403) |
| M/Lopsin | Rabbit | 1:500 | Millipore (AB5405) |

**Supplementary Table 3.** List of primers used for targeted deep sequencing

|  | **PCR** | |
| --- | --- | --- |
| **Target gene** | **Forward primer (5’ to 3’)** | **Reverse primer (5’ to 3’)** |
| *VEGFA* (human) | ACACTCTTTCCCTACACGACGCTCTTCCGATCTGCCTCTCATGCAGTGGTGAA | GTGACTGGAGTTCAGACGTGTGCTCTTCCGATCTCCACCTGCATGGTGATGTTG |
| *Vegfa* (mouse) | ACACTCTTTCCCTACACGACGCTCTTCCGATCTCCCACACAGTGATCAAGTTC | GTGACTGGAGTTCAGACGTGTGCTCTTCCGATCTCTTCATCGTTACAGCAGCCTG |
| *AAVS1* (human) | ACACTCTTTCCCTACACGACGCTCTTCCGATCTGACCACCTTATATTCCCAGGG | GTGACTGGAGTTCAGACGTGTGCTCTTCCGATCTGTGGGGGTTAGACCCAATATC |
| *Rosa26* (mouse) | ACACTCTTTCCCTACACGACGCTCTTCCGATCTATCAGTAAGGGAGCTGCAGTG | GTGACTGGAGTTCAGACGTGTGCTCTTCCGATCTCAGAAGACTCCCGCCCATC |
| *Vegfa* (mouse) off target 1 | ACACTCTTTCCCTACACGACGCTCTTCCGATCTGTGATCAGCTGACTTCCAGTTC | GTGACTGGAGTTCAGACGTGTGCTCTTCCGATCTCTCCACAACTCAAGTCCCATTAC |

Adaptor sequence

Forward primer: ACACTCTTTCCCTACACGACGCTCTTCCGATCT

Reverse primer: GTGACTGGAGTTCAGACGTGTGCTCTTCCGATCT

Target sequences of *Vegfa* (mouse) off target 1: CTCCTGGAAGATtTtCACCA **GGG** (gene: intergenic region)
